# Sex and race differences in the performance of the European Society of Cardiology 0/1‐h algorithm with high‐sensitivity troponin T

**DOI:** 10.1002/clc.24199

**Published:** 2023-12-13

**Authors:** Michael W. Supples, Anna C. Snavely, James C. O'Neill, Nicklaus P. Ashburn, Brandon R. Allen, Robert H. Christenson, Richard Nowak, R. Gentry Wilkerson, Bryn E. Mumma, Troy Madsen, Jason P. Stopyra, Simon A. Mahler

**Affiliations:** ^1^ Department of Emergency Medicine Wake Forest School of Medicine Winston‐Salem North Carolina USA; ^2^ Department of Biostatistics and Data Science Wake Forest School of Medicine Winston‐Salem North Carolina USA; ^3^ Section on Cardiovascular Medicine, Department of Internal Medicine Wake Forest School of Medicine Winston‐Salem North Carolina USA; ^4^ Department of Emergency Medicine University of Florida College of Medicine Gainesville Florida USA; ^5^ Department of Pathology University of Maryland School of Medicine Baltimore Maryland USA; ^6^ Department of Emergency Medicine Henry Ford Health System Detroit Michigan USA; ^7^ Department of Emergency Medicine University of Maryland School of Medicine Baltimore Maryland USA; ^8^ Department of Emergency Medicine University of California Davis School of Medicine Sacramento California USA; ^9^ Division of Emergency Medicine University of Utah School of Medicine Salt Lake City Utah USA; ^10^ Department of Epidemiology and Prevention Wake Forest School of Medicine Winston‐Salem North Carolina USA; ^11^ Department of Implementation Science Wake Forest School of Medicine Winston‐Salem North Carolina USA

## Abstract

The diagnostic performance of the high‐sensitivity troponin T (hs‐cTnT) European Society of Cardiology (ESC) 0/1‐h algorithm in sex and race subgroups of US Emergency Department (ED) patients is unclear. A pre‐planned subgroup analysis of the STOP‐CP cohort study was conducted. Participants with 0‐ and 1‐h hs‐cTnT measures from eight US EDs (1/2017 to 9/2018) were stratified into rule‐out, observation, and rule‐in zones using the hs‐cTnT ESC 0/1 algorithm. The primary outcome was adjudicated 30‐day cardiac death or MI. The proportion with the primary outcome in each zone was compared between subgroups with Fisher's exact tests. The negative predictive value (NPV) of the ESC 0/1 rule‐out zone for 30‐day CDMI was calculated and compared between subgroups using Fisher's exact tests. Of the 1422 patients enrolled, 54.2% (770/1422) were male and 58.1% (826/1422) white with a mean age of 57.6 ± 12.8 years. At 30 days, cardiac death or myocardial infarction (MI) occurred in 12.9% (183/1422) of participants. Among patients stratified to the rule‐out zone, 30‐day cardiac death or MI occurred in 1.1% (5/436) of women versus 2.1% (8/436) of men (*p* = .40) and 1.2% (4/331) of non‐white patients versus 1.8% (9/490) of white patients (*p* = .58). The NPV for 30‐day cardiac death or MI was similar among women versus men (98.9% [95% confidence interval, CI: 97.3–99.6] vs. 97.9% [95% CI: 95.9–99.1]; *p* = .40) and among white versus non‐white patients (98.8% [95% CI: 96.9–99.7] vs. 98.2% [95% CI: 96.5–99.2]; *p* = .39). NPVs <99% in each subgroup suggest the hs‐cTnT ESC 0/1‐h algorithm may not be safe for use in US EDs. Trial Registration: High‐Sensitivity Cardiac Troponin T to Optimize Chest Pain Risk Stratification (STOP‐CP; ClinicalTrials.gov: NCT02984436; https://clinicaltrials.gov/ct2/show/NCT02984436).

## Graphical abstract

Primary outcome: Negative predictive value (NPV) for 30‐day cardiac death or myocardial infarction among sex and race subgroups of the STOP‐CP Study.

Women NPV: 97.9% (95% confidence interval [CI]: 95.9–99.1).

Men NPV: 98.9% (95% CI: 97.3‐99.6).

Non‐white patients NPV: 98.2% (95% CI: 96.5–99.2).

White patients NPV: 98.8% (95% CI: 96.9–99.7).

Conclusion: The ESC 0/1‐h hs‐cTnT rule failed to achieve the commonly accepted NPV safety threshold of 99% for all subgroups.

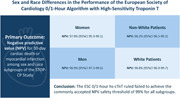

## 1 INTRODUCTION

Chest pain leads to over 6.5 million Emergency Department (ED) visits in the United States per year.^1^, ^2^, ^3^ To optimize care for these patients, accelerated diagnostic protocols (ADPs) have been developed to objectively guide patient risk stratification. The high‐sensitivity cardiac troponin T European Society of Cardiology (ESC) 0/1‐h algorithm (hs‐cTnT ESC 0/1‐h algorithm) is a commonly used ADP and is recommended by European and US guidelines.^4^, ^5^, ^6^, ^7^, ^8^, ^9^, ^10^

In several studies, mostly conducted in Europe and Australasia, the hs‐cTnT ESC 0/1‐h algorithm has demonstrated a ≥99% negative predictive value (NPV) for 30‐day myocardial infarction (MI) or cardiac death.^6^, ^7^, ^11^, ^12^ Its performance in US patients with acute chest pain is not as well established.^12^ In the primary analysis of the STOP‐CP multisite US cohort, the hs‐cTnT ESC 0/1‐h algorithm did not achieve a 99% NPV, which is the threshold commonly used for ADP safety.^13^, ^14^ However, it is unclear whether diagnostic performance was consistent across key patient subgroups, such as men, women, white patients, and non‐white patients and there is a paucity of prior data evaluating sex or race differences in algorithm performance. Prior studies have demonstrated significant differences in 99th percentile hs‐cTnT values among men versus women and white patients versus non‐white patients.^15^, ^16^, ^17^, ^18^ In addition, it has been hypothesized that greater racial diversity of US ED patients may explain differences in the diagnostic performance of the ESC 0/1‐h algorithm in the United States versus Europe and Australasia. Thus, it is plausible that the performance of the ESC 0/1‐h algorithm may differ based on demographics.

The primary objective of this pre‐planned STOP‐CP subgroup analysis was to evaluate and compare the diagnostic performance of the hs‐cTnT ESC 0/1‐h algorithm for 30‐day cardiac death or MI in men versus women and white versus nonwhite patients within the STOP‐CP cohort. A secondary objective was to evaluate and compare diagnostic performance for 30‐day major adverse cardiovascular events (MACEs; defined as cardiac death, MI, or coronary revascularization) in these subgroups. In addition, we evaluate whether combining the hs‐cTnT ESC 0/1‐h algorithm with the History, ECG, Age, and Risk factors (HEAR) score can improve test characteristics among patients in each subgroup.^19^

## 2 METHODS

### 2.1 Study design and setting

This is a preplanned subgroup analysis of the High‐Sensitivity Cardiac Troponin T (Gen 5 STAT assay) to Optimize Chest Pain Risk Stratification (STOP‐CP; ClinicalTrials. gov: NCT02984436) prospective, multicenter cohort study. STOP‐CP enrolled patients with symptoms concerning for ACS at eight US EDs from January 25, 2017, to June 9, 2018. Study sites included the University of Florida, Gainesville, Florida; Wake Forest University, Winston Salem, North Carolina; Henry Ford Health System, Detroit, Michigan; University of Maryland St. Joseph Medical Center, Towson, Maryland; University of Maryland Medical Center, Baltimore, Maryland; University of Maryland Baltimore Washington Medical Center, Glen Burnie, Maryland; University of California‐Davis, Davis, California; and University of Utah, Salt Lake City, Utah. Institutional review board approval was obtained at all sites. Written informed consent was obtained for enrollment. STOP‐CP methods are previously described.^14^ The Standards for Reporting of Diagnostic Accuracy Studies (STARD) guidelines helped direct the research and manuscript development processes.^20^

### 2.2 Study population

We prospectively enrolled ED patients ≥21 years of age with serial troponins ordered for the evaluation of possible acute coronary syndrome (ACS). Exclusion criteria included ST‐elevation myocardial infarction, systolic blood pressure <90 mmHg, life expectancy <90 days, a noncardiac illness requiring admission, inability to provide consent or be contacted for follow‐up, non‐English speaking, pregnancy, being a prisoner, or previous enrollment in the study.

### 2.3 Data collection

Serial blood samples were collected for hs‐cTnT measurement at baseline (<1 h from the first clinical blood draw) and 1‐h later in lithium heparin tubes. hs‐cTnT was quantified with the Gen 5 STAT assay on the Cobas e 601 analyzer (Roche Diagnostics). The assay has a range of 3–10,000 ng/L, limit of quantification at 6 ng/L, and a 99th percentile upper reference limit (URL) of 19 ng/L in the United States with a coefficient of variation of <10%.^21^ Treating providers were blinded to hs‐cTnT results; therefore, patient care was dictated by local standards of care and guided by contemporary cTn results.

Demographic data were collected by research staff from the patient by self‐report and were supplemented by the patient's electronic medical record. These included age on the day of emergency department visit, sex, race and ethnicity, and risk factors (current or prior tobacco use, hypertension, hyperlipidemia, diabetes, family history of coronary artery disease [CAD], obesity, prior cerebrovascular accident, peripheral vascular disease, and end‐stage renal disease). Initial electrocardiogram (ECG) findings of acute ischemia were indicated by the treating physician.

### 2.4 ESC 0/1‐h algorithm

In each patient, hs‐cTnT measures were used to stratify patients into rule‐out, observation, and rule‐in zones using established assay‐specific cut‐points.^4^, ^5^, ^14^ However, the hs‐cTnT ESC 0/1‐h algorithm's 0‐h rule‐out cut‐point of 5 ng/L (the limit of detection) was modified to 6 ng/L (the limit of quantification), because the U.S. Food and Drug Administration does not allow reporting below the limit of quantification. Based on prior derivation and validation studies, patients stratified to the rule‐out zone were expected to have ≥99% NPV for cardiac death or MI.^6^, ^7^, ^8^, ^22^ In addition, we evaluated the combination of the hs‐cTnT ESC 0/1‐h algorithm with a HEAR score within each subgroup. HEAR scores were determined prospectively by the patient's treating provider. Consistent with prior studies, a score of 0–3 was considered low‐risk, 4–6 was moderate‐risk, and scores ≥7 were high‐risk.^19^

### 2.5 Outcomes

The primary outcome was 30‐day cardiac death or MI, inclusive of index visit events. Secondary outcomes included: (1) 30‐day MACE, (2) the individual MACE components (cardiac death, MI, and coronary revascularization) at index and from index through 30 days, and (3) efficacy, defined as the proportion of patients classified into the rule‐out zone.^23^, ^24^ Medical record review and telephone follow‐up through 30 days were completed to determine outcomes. Expert reviewers adjudicated any patient who experienced death, had a clinical diagnosis of MI, or had an elevated contemporary cTn. Expert adjudicators (M.H.i.V., M.M., J.P.S., and J.M.) classified deaths as cardiac or noncardiac based on the Action to Control Cardiovascular Risk in Diabetes (ACCORD) trial definition, except for death due to stroke which was classified as a noncardiac death.^25^ If the cause of death could not be determined, it was considered cardiac. MI was determined by the Fourth Universal Definition of MI: rise and fall of troponin (with at least one value ˃99th percentile URL) with symptoms of ischemia, ECG evidence of ischemia, imaging evidence of new nonviable myocardium, a new regional wall motion abnormality, or evidence of thrombus on angiography.^26^ Adjudicators had access to all clinical data, including the local clinical contemporary troponin assay results (Table S1), but were blinded to the hs‐cTnT results. Any discrepancies between adjudicators were resolved through review by a third adjudicator.

### 2.6 Statistical analysis

Counts, percentages, means, and standard deviations, or medians and interquartile (IQR) ranges were used to describe the study population. To evaluate the performance of the ESC 0/1‐h algorithm, sensitivity, specificity, NPV and PPV, and negative and positive likelihood ratios (−LR and +LR) for 30‐day cardiac death or MI and 30‐day MACE were calculated. For efficacy, sensitivity, specificity, NPV, and PPV, exact 95% confidence intervals (95% CI) were computed, and Fisher's exact tests were used to compare by sex (men vs. women) and race (white vs. nonwhite patients). Likelihood ratios were calculated and reported with 95% CIs using the method of Simel et al.^27^ The asymptotic hypothesis test developed by Luts et al. was used to compare LRs by sex and race.^28^ Consistent with prior studies, sensitivity, NPV, and −LR were calculated for the rule‐out zone (i.e., rule‐out vs rule‐in or observation) and specificity, PPV, and +LR were calculated for the rule‐in zone (i.e., rule‐in vs. observation or rule‐out).^6^, ^7^, ^14^, ^19^, ^29^ Fisher's exact tests were used to compare cardiac death or MI and MACE at index and from index though 30‐days among sexes and races.

To assess the association of sex or race with index and 30‐day cardiac death or MI and 30‐day MACE, multivariable logistic regression was performed. Models were adjusted for age, sex, race (white vs. nonwhite patients), hypertension, diabetes, hyperlipidemia, obesity (BMI ≥ 30 kg/m^2^), current smoking, prior stroke, peripheral vascular disease, and end‐stage renal disease. These variables were selected due to their relevance and inclusion in previous cardiovascular risk stratification work.^29^ Ethnicity was not included in this analysis. For each outcome, we computed two separate adjusted models, one containing an interaction term between ESC 0/1‐h zone and sex, and another containing an interaction term between ESC 0/1‐h zone and race. Separate, unadjusted logistic regression models were fit to evaluate the association between sex and race with the primary and secondary outcomes within each ESC 0/1‐h zone. Unadjusted or adjusted odds ratios (aORs) with corresponding 95% CIs were calculated appropriately for each logistic model.

In addition, we evaluated the diagnostic performance of the combination of the hs‐cTnT ESC 0/1‐h algorithm with a HEAR score. For this combination, patients were classified to the rule‐out zone only if they met both the ESC 0/1‐h algorithm rule‐out cut‐points and had a low‐risk HEAR score of 0‐3. Patients with a HEAR score of ≥7 were classified to the rule‐in zone regardless of hs‐cTnT measures. Patients meeting the ESC 0/1‐h algorithm's rule‐out criteria, who had a HEAR score of 4–6, were reclassified to the observation zone.

## 3 RESULTS

This preplanned subgroup analysis included 1422 patients. The patient flow diagram is shown in Figure S1. The cohort was 54.1% (770/1422) male and 58.1% (826/1422) white, with a mean age of 57.6 ± 12.8 years. Patient demographics are presented in Table 1. The rate of 30‐day cardiac death or MI in the cohort was 12.9% (183/1422), occurring in 8.7% (57/652) of women compared to 16.4% (126/770) of men (*p* < .001) and 11.2% (67/596) of nonwhite patients compared to 14.0% (116/826) of white patients (*p* = .12). MACE at 30 days was also more common in men than women (18.2% [140/770] vs. 9.7% [63/652]; *p* < .001) and among white patients than nonwhite patients (16.0% [132/826] vs. 11.9% [71/596]; *p* = .03). Description of each patient classified to the rule‐out zone who experienced cardiac death or MI is available in Table S2. Event rates using all‐cause death instead of cardiac death are available in Table S3.

**Table 1:** Cohort characteristics.

	Female, *n* = 652, *n* (%)	Male, *n* = 770, *n* (%)	Nonwhite, *n* = 596, *n* (%)	White, *n* = 826, *n* (%)	Total *N* = 1422
Age, mean (SD), years	58.4 (12.8)	56.9 (12.8)	55.2 (12.1)	59.3 (13.0)	57.6 (12.8)
Sex					
Male	0 (0)	770 (100)	300 (50.3)	470 (56.9)	770 (54.2)
Race					
American Indian/Alaska Native	9 (1.4)	14 (1.8)	23 (3.9)	0 (0)	23 (1.6)
Asian	6 (0.9)	6 (0.8)	12 (2.0)	0 (0)	12 (0.8)
Native Hawaiian	0 (0)	2 (0.3)	2 (0.3)	0 (0)	2 (0.1)
Black or African American	268 (41.1)	256 (33.2)	524 (87.9)	0 (0)	524 (36.8)
White	356 (54.6)	470 (61.0)	0 (0)	826 (100)	826 (58.1)
Other	13 (2.0)	22 (2.9)	35 (5.9)	0 (0)	35 (2.5)
Ethnicity					
Hispanic or Latino	23 (3.5)	29 (2.8)	33 (5.5)	19 (2.3)	52 (3.7)
Not Hispanic or Latino	635 (95.9)	732 (95.1)	556 (93.3)	801 (97.0)	1357 (95.4)
Unknown	4 (0.6)	9 (1.2)	7 (1.2)	6 (0.7)	13 (0.9)
Risk factors					
Current or history of smoking	318 (48.8)	469 (61.0)	342 (57.5)	445 (53.9)	787 (55.4)
Hypertension	427 (65.5)	521 (67.7)	438 (73.5)	510 (61.7)	948 (66.7)
Hyperlipidemia	287 (44.0)	391 (50.8)	280 (47.0)	398 (48.2)	678 (47.7)
Diabetes	198 (30.4)	217 (28.2)	205 (34.4)	210 (25.4)	415 (29.2)
Family history of coronary disease	325 (49.8)	333 (43.2)	251 (42.1)	407 (49.3)	658 (46.3)
BMI ≥ 30 kg/m^2^	363 (55.7)	382 (49.7)	318 (53.4)	427 (51.8)	745 (52.4)
Prior cerebrovascular accident	63 (9.7)	91 (11.8)	70 (11.7)	84 (10.2)	154 (10.8)
Prior peripheral vascular disease	37 (5.7)	52 (6.8)	38 (6.4)	51 (6.2)	89 (6.3)
Prior end‐stage renal disease	30 (4.6)	41 (5.3)	40 (6.7)	31 (3.8)	71 (5.0)
Chest pain onset					
≤3 h from arrival	225 (34.5)	277 (36.0)	195 (32.7)	307 (37.2)	502 (35.3)
˃3 h from arrival	423 (65.3)	489 (63.5)	400 (67.2)	512 (62.5)	912 (64.5)
ECG at arrival					
Ischemic	37 (5.7)	51 (6.6)	48 (8.1)	40 (4.8)	88 (6.2)
Nonischemic	615 (94.3)	719 (93.4)	548 (91.9)	786 (95.2)	1334 (93.8)
Initial study hs‐cTnT sample, median (IQR), ng/L	7 (4–15)	11 (6–27)	10 (5–23)	9 (5–20)	9 (5–21)

Abbreviations: BMI, body mass index; ECG, electrocardiogram; hs‐cTnT, high sensitivity troponin T; IQR, interquartile range; SD, standard deviation.

### 3.1 ESC 0/1‐h algorithm by sex

The efficacy of the hs‐cTnT ESC 0/1‐h algorithm was higher in women than men, with 66.9% (436/652) of women stratified to the rule‐out zone compared to 50.0% (385/770) of men (*p* < .001). Among patients stratified into the rule‐out zone, 30‐day cardiac death or MI occurred in 1.1% (5/436) of women versus 2.1% (8/385) of men (OR: 0.55, 95% CI: 0.16–1.65). MACE at 30 days did not significantly differ by sex among those in the rule‐out zone, with a MACE rate of 1.8% (8/436) for women and 3.9% (15/385) for men (OR: 0.46, 95% CI: 0.18–1.07). The NPV for 30‐day cardiac death or MI was 98.9% (95% CI: 97.3–99.6) in women and 97.9% (95% CI: 95.9–99.1) in men (*p* = .40). Events for men and women stratified by ESC 0/1‐h rule‐out, observation, and rule‐in zones are summarized in Table 2.

**Table 2:** Safety events among females and males by ESC 0/1 zone (unadjusted).

Rule‐out	Female, *n* = 436, *n* (%)	Male, *n* = 385, *n* (%)	Total, *n* = 821, *n* (%)	Odds ratio (95% CI)
Index				
Cardiac death	1 (0.2)	0 (0)	1 (0.1)	NA
MI	4 (0.9)	4 (1)	8 (1)	0.88 (0.21–3.75)
Revascularization	2 (0.5)	8 (2.1)	10 (1.2)	**0.22 (0.03–0.87)**
Cardiac death or MI	5 (1.1)	4 (1)	9 (1.1)	1.1 (0.29–4.49)
MACE	7 (1.6)	9 (2.3)	16 (1.9)	0.68 (0.24–1.85)
30‐day (index + follow‐up)				
Cardiac death	1 (0.2)	0 (0)	1 (0.1)	NA
MI	4 (0.9)	8 (2.1)	12 (1.5)	0.44 (0.12–1.4)
Revascularization	3 (0.7)	10 (2.6)	13 (1.6)	**0.26 (0.06–0.86)**
Cardiac death or MI	5 (1.1)	8 (2.1)	13 (1.6)	0.55 (0.16–1.65)
MACE	8 (1.8)	15 (3.9)	23 (2.8)	0.46 (0.18–1.07)

**Observation**	Female, *n* = 159, *n* (%)	Male, *n* = 252, *n* (%)	Total, *N* = 411, *n* (%)	Odds ratio (95% CI)
Index				
Cardiac death	0 (0)	0 (0)	0 (0)	NA
MI	13 (8.2)	38 (15.1)	51 (12.4)	**0.5 (0.25–0.95)**
Revascularization	4 (2.5)	16 (6.3)	20 (4.9)	0.38 (0.11–1.06)
Cardiac death or MI	13 (8.2)	38 (15.1)	51 (12.4)	**0.5 (0.25–0.95)**
MACE	13 (8.2)	42 (16.7)	55 (13.4)	**0.45 (0.22–0.84)**
30‐day (index + follow‐up)				
Cardiac death	1 (0.6)	1 (0.4)	2 (0.5)	NA
MI	15 (9.4)	42 (16.7)	57 (13.9)	**0.52 (0.27–0.95)**
Revascularization	8 (5)	21 (8.3)	29 (7.1)	0.58 (0.24–1.3)
Cardiac death or MI	16 (10.1)	43 (17.1)	59 (14.4)	**0.54 (0.29–0.98)**
MACE	19 (11.9)	50 (19.8)	69 (16.8)	**0.55 (0.3–0.96)**

Rule‐in	Female, *n* = 57, *n* (%)	Male, *n* = 133, *n* (%)	Total, *N* = 190, *n* (%)	Odds ratio (95% CI)
Index				
Cardiac death	0 (0)	1 (0.8)	1 (0.5)	NA
MI	35 (61.4)	70 (52.6)	105 (55.3)	1.43 (0.76–2.72)
Revascularization	13 (22.8)	25 (18.8)	38 (20)	1.28 (0.59–2.69)
Cardiac death or MI	35 (61.4)	70 (52.6)	105 (55.3)	1.43 (0.76–2.72)
MACE	35 (61.4)	70 (52.6)	105 (55.3)	1.43 (0.76–2.72)
30‐day (index + follow‐up)				
Cardiac death	0 (0)	6 (4.5)	6 (3.2)	NA
MI	36 (63.2)	73 (54.9)	109 (57.4)	1.41 (0.75–2.69)
Revascularization	15 (26.3)	28 (21.1)	43 (22.6)	1.34 (0.64–2.73)
Cardiac death or MI	36 (63.2)	75 (56.4)	111 (58.4)	1.33 (0.7–2.54)
MACE	36 (63.2)	75 (56.4)	111 (58.4)	1.33 (0.7–2.54)

Abbreviations: CI, confidence interval; ESC, European Society of Cardiology; MACE, major adverse cardiovascular event; MI, myocardial infarction.

The ESC 0/1‐h algorithm classified 8.7% (57/652) of women into the rule‐in zone versus 17.3% of men (133/770) (*p* < .001). Among rule‐in patients, the rate of index cardiac death or MI was similar between women and men (61.4% [35/57] vs. 52.6% [70/133]; OR: 1.43, 95% CI 0.76–2.72). The PPV for 30‐day cardiac death or MI was 63.2% (95% CI: 49.3–75.6) for women and 56.4% (95% CI: 47.5–65.0) for men (*p* = .42). However, the +LR for 30‐day cardiac death or MI was higher for women than men (17.9, 95% CI: 11.3–28.5 vs. 6.6, 95% CI: 5.0–8.8; *p* < .001). The diagnostic performance of the algorithm by sex is summarized in Table S4 and Figure S1. Diagnostic performance using all‐cause death instead of cardiac death is provided in Table S5.

**Figure 1:**
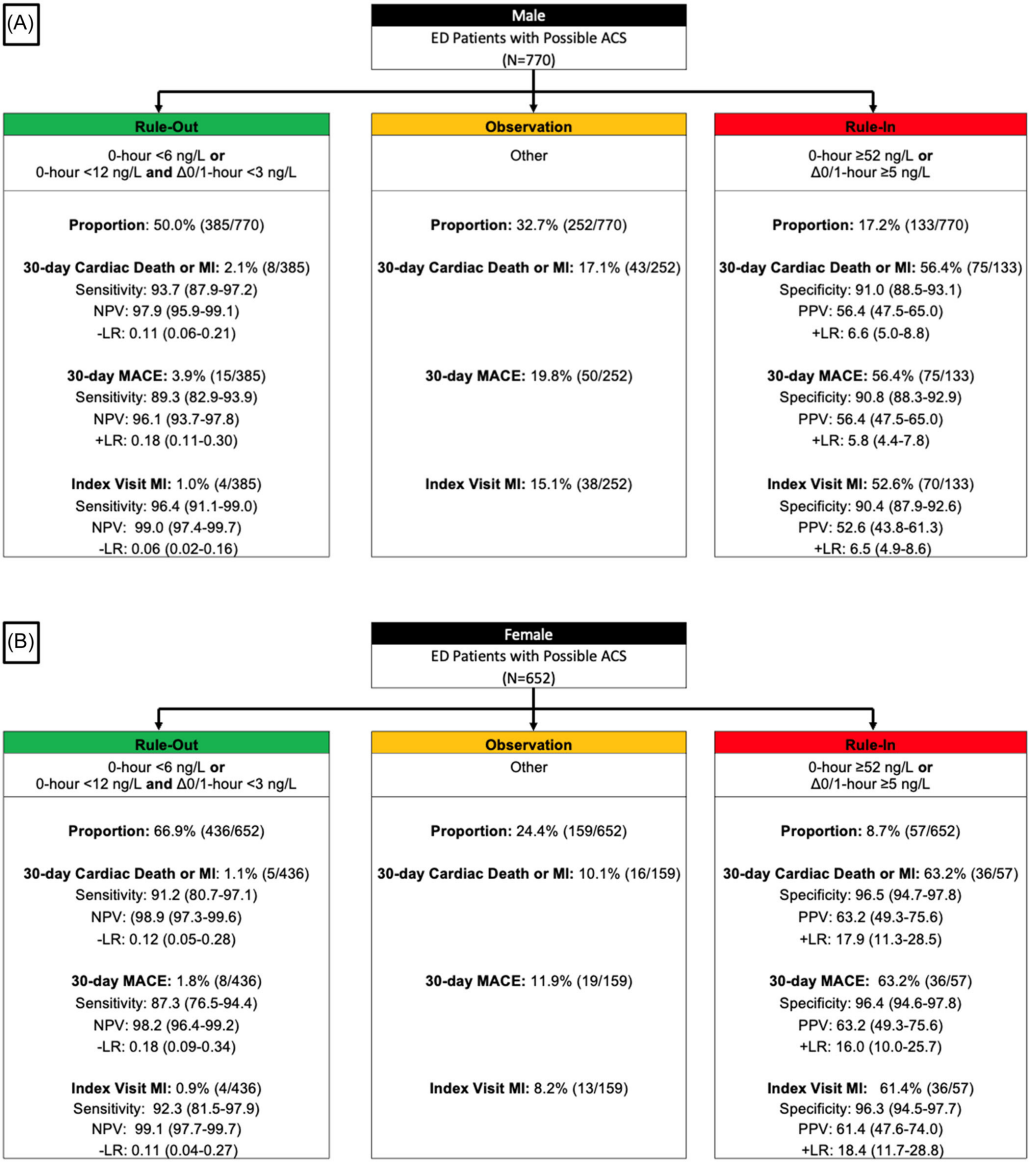
(A, B) The ESC 0/1‐h algorithm among male and female patients for 30‐day cardiac death or MI, and 30‐day MACE. (A) Male, (B) Female. ACS, acute coronary syndrome; CAD, coronary artery disease; ED, emergency department; ESC, European Society of Cardiology; MACE, major adverse cardiovascular event; MI, myocardial infarction; NPV, negative predictive value; PPV, positive predictive value.

The interaction between the ESC 0/1‐h algorithm and sex was significant for 30‐day cardiac death or MI (*p* = .04) and 30‐day MACE (*p* = .021). The adjusted odds of 30‐day cardiac death or MI and 30‐day MACE were higher for men in the rule‐out and observation zones based on point estimates, but higher for women in the rule‐in zone. Figure S2 and Table S6 show the aORs for index and 30‐day cardiac death or MI and MACE (Figure 1).

### 3.2 ESC 0/1‐h algorithm by race

**Figure 2:**
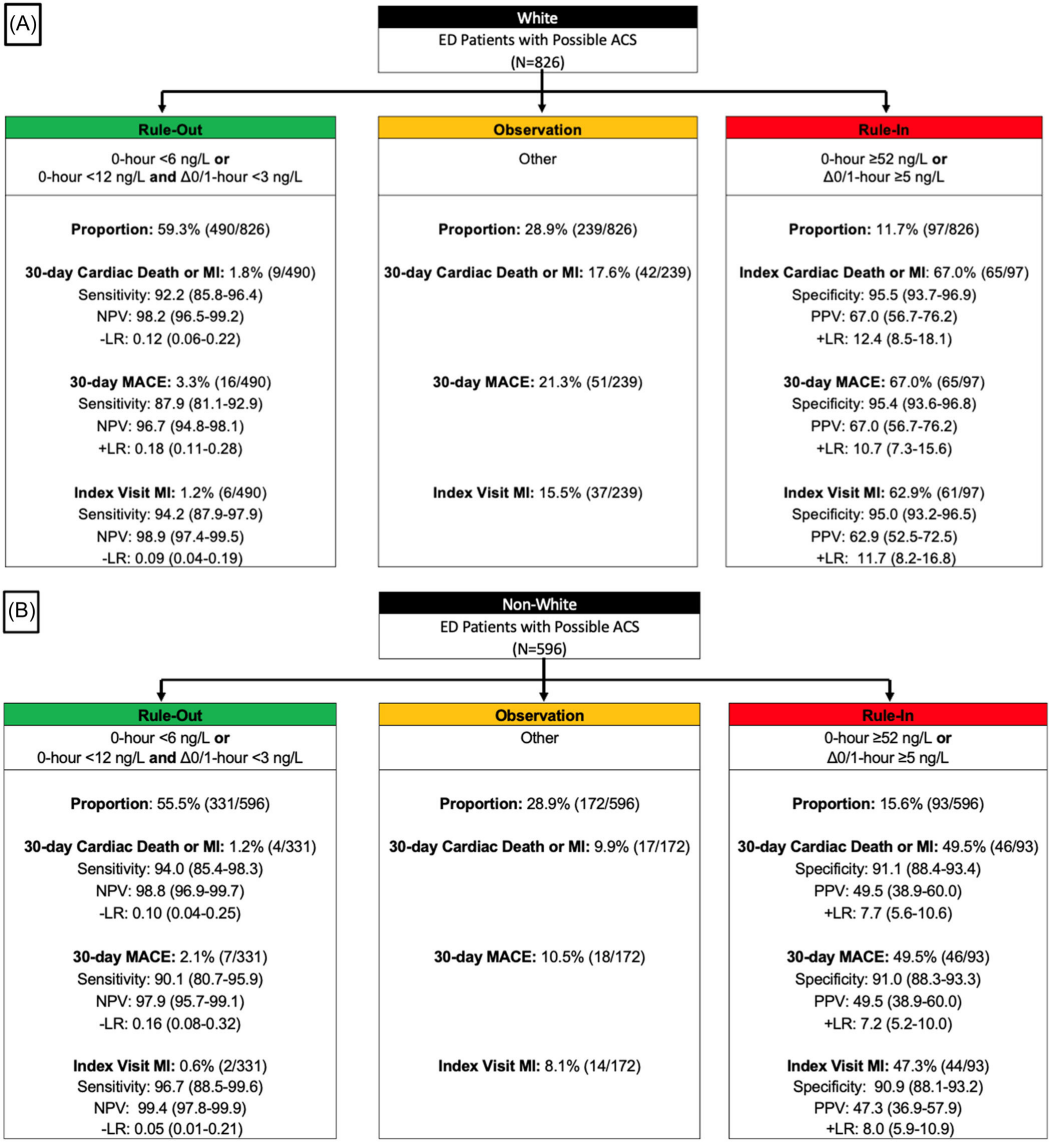
(A, B) The ESC 0/1‐h algorithm among white and nonwhite patients for 30‐day cardiac death or MI and 30‐day MACE. (A) White, (B) Nonwhite. ACS, acute coronary syndrome; CAD, coronary artery disease; ED, emergency department; ESC, European Society of Cardiology; LR, likelihood ratio; MACE, major adverse cardiovascular event; MI, myocardial infarction; NPV, negative predictive value; PPV, positive predictive value.

The efficacy of the ESC 0/1‐h algorithm was similar between nonwhite and white patients (55.5% [331/596] vs. 59.3% [490/826]; *p* = .16]. Among patients who were ruled out by ESC 0/1‐h algorithm, cardiac death or MI at 30 days occurred in 1.2% (4/331) of nonwhite patients compared to 1.8% (9/490) of white patients (OR: 0.65, 95% CI: 0.18–2.03). MACE at 30 days was also similar by race among patients classified into the rule‐out zone, with a MACE rate of 2.1% (7/331) for nonwhite patients compared to 3.3% (16/490) for white patients (OR: 0.64, 95% CI: 0.24–1.52). The NPV for 30‐day cardiac death or MI was 98.8% (95% CI: 96.9–99.7) for nonwhite patients and 98.2% (96.5–99.2) for white patients (*p* = .58). Event rates for nonwhite and white patients stratified by ESC 0/1‐h algorithm rule‐out, observation, and rule‐in zones are summarized in Table 3 and Figure 2.

**Table 3:** Safety events among nonwhite and white patients by ESC 0/1 zone (unadjusted).

Rule‐out	Nonwhite, *n* = 331, *n* (%)	White, *n* = 490, *n* (%)	Total, *n* = 821, *n* (%)	Odd ratio (95% CI)
Index				
Cardiac death	0 (0)	1 (0.2)	1 (0.1)	NA
MI	2 (0.6)	6 (1.2)	8 (1)	0.49 (0.07–2.14)
Revascularization	1 (0.3)	9 (1.8)	10 (1.2)	**0.16 (0.01–0.87)**
Cardiac death or MI	2 (0.6)	7 (1.4)	9 (1.1)	0.42 (0.06–1.75)
MACE	3 (0.9)	13 (2.7)	16 (1.9)	0.34 (0.08–1.05)
30‐day (index + follow‐up)				
Cardiac death	0 (0)	1 (0.2)	1 (0.1)	NA
MI	4 (1.2)	8 (1.6)	12 (1.5)	0.74 (0.2–2.36)
Revascularization	3 (0.9)	10 (2)	13 (1.6)	0.44 (0.1–1.45)
Cardiac death or MI	4 (1.2)	9 (1.8)	13 (1.6)	0.65 (0.18–2.03)
MACE	7 (2.1)	16 (3.3)	23 (2.8)	0.64 (0.24–1.52)

Observation	Nonwhite, *n* = 172, *n* (%)	White, *n* = 239, *n* (%)	Total, *N* = 411, *n* (%)	Odd ratio (95% CI)
Index				
Cardiac death	0 (0)	0 (0)	0 (0)	NA
MI	14 (8.1)	37 (15.5)	51 (12.4)	**0.48 (0.25–0.91)**
Revascularization	4 (2.3)	16 (6.7)	20 (4.9)	**0.33 (0.09–0.92)**
Cardiac death or MI	14 (8.1)	37 (15.5)	51 (12.4)	**0.48 (0.25–0.91)**
MACE	14 (8.1)	41 (17.2)	55 (13.4)	**0.43 (0.22–0.79)**
30‐day (index + follow‐up)				
Cardiac death	0 (0)	2 (0.8)	2 (0.5)	NA
MI	17 (9.9)	40 (16.7)	57 (13.9)	**0.55 (0.29–0.98)**
Revascularization	7 (4.1)	22 (9.2)	29 (7.1)	**0.42 (0.16–0.96)**
Cardiac death or MI	17 (9.9)	42 (17.6)	59 (14.4)	**0.51 (0.28–0.92)**
MACE	18 (10.5)	51 (21.3)	69 (16.8)	**0.43 (0.24–0.76)**

Rule‐in	Nonwhite, *n* = 93, *n* (%)	White, *n* = 97, *n* (%)	Total, *N* = 190, *n* (%)	Odd ratio (95% CI)
Index				
Cardiac death	0 (0)	1 (1)	1 (0.5)	NA
MI	44 (47.3)	61 (62.9)	105 (55.3)	**0.53 (0.3–0.94)**
Revascularization	16 (17.2)	22 (22.7)	38 (20)	0.71 (0.34–1.45)
Cardiac death + MI	44 (47.3)	61 (62.9)	105 (55.3)	**0.53 (0.3–0.94)**
MACE	44 (47.3)	61 (62.9)	105 (55.3)	**0.53 (0.3–0.94)**
30‐day (index + follow‐up)				
Cardiac death	2 (2.2)	4 (4.1)	6 (3.2)	0.51 (0.07–2.68)
MI	45 (48.4)	64 (66)	109 (57.4)	**0.48 (0.27–0.86)**
Revascularization	18 (19.4)	25 (25.8)	43 (22.6)	0.69 (0.34–1.37)
Cardiac death + MI	46 (49.5)	65 (67)	111 (58.4)	**0.48 (0.27–0.86)**
MACE	46 (49.5)	65 (67)	111 (58.4)	**0.48 (0.27–0.86)**

Abbreviations: CI, confidence interval; ESC, European Society of Cardiology; MACE, major adverse cardiovascular event; MI, myocardial infarction.

The ESC 0/1‐h algorithm classified 15.6% (93/596) of nonwhite patients into the rule‐in zone versus 11.7% (97/826) of white patients (*p* = .04). Among rule‐in patients, the rate of index cardiac death or MI was lower in nonwhite patients than white patients (47.3% [44/93] vs. 62.9% [61/97]; OR: 0.53, 95% CI: 0.30–0.94). At 30 days, 49.5% (46/93) of nonwhite patients experienced cardiac death or MI compared to 67.0% (65/97) of white patients (OR: 0.48, 95% CI: 0.27–0.86). The PPV for 30‐day cardiac death or MI was 49.5% (95% CI: 38.9–60.0) for nonwhite patients and 67.0% (95% CI: 56.7–76.2) for white patients (*p* = .21). The test characteristics for the rule‐in zone are presented in Table 3. The diagnostic performance of the algorithm by race is summarized in Tables S4 and S5 and Figure S2.

The overall interaction of the ESC 0/1‐h algorithm with race was not significant for outcomes of 30‐day cardiac death or MI (*p* = .951) or 30‐day MACE (*p* = .861). Adjusted odds ratio point estimates remained greater than one for white patients compared to nonwhite patients in each ESC 0/1‐h algorithm zone. Figure S2 and Table S6 show the aORs for index and 30‐day cardiac death or MI and MACE.

### 3.3 Combination of ESC 0/1‐h algorithm with the HEAR score

NPV was increased by adding the HEAR score to the hs‐cTnT ESC 0/1‐h algorithm in each subgroup. For the primary outcome of 30‐day cardiac death or MI, the NPV of the combination was 99.6% (95% CI: 97.6%–100%) for females, 99.0% (95% CI: 96.5–99.9) for males, 99.5% (95% CI: 97.1%–100%) for non‐whites, and 99.2% (95% CI: 97.2%–99.9%) for whites. However, even when the hs‐cTnT ESC algorithm was combined with the HEAR score, the NPV for 30‐day MACE remained below 99.0% for all subgroups. See Table S7 for full test characteristics among subgroups. The diagnostic performance of the ESC 0/1‐h algorithm with the HEAR score using all‐cause death instead of cardiac death is provided in Table S5.

## 4 DISCUSSION

In this multicenter, prospective study, the hs‐cTnT ESC 0/1‐h algorithm failed to achieve an acceptable^13^ NPV ≥99% for 30‐day cardiac death or MI among any sex or race subgroup. Although efficacy was higher in women, there was no difference in safety among men versus women. While fewer women were placed in the rule‐in zone, they were more likely to experience 30‐day cardiac death or MI or MACE than men. Efficacy and safety were similar among white and nonwhite patients. More nonwhite patients were classified to the rule‐in zone, but they had lower odds of 30‐day cardiac death or MI than white patients in the rule‐in zone.

Use of the hs‐cTnT ESC 0/1‐h algorithm for evaluation of ED patients with chest pain is a class I recommendation by the ESC guidelines.^4^ This recommendation is based on international studies of the algorithm, which have demonstrated an NPV ≥99% for index‐MI,^30^, ^31^ 30‐day cardiac death or MI,^32^ or 30‐day MACE.^6^, ^7^ However, in some cohorts, the hs‐cTnT ESC 0/1‐h algorithm has failed to achieve the ≥99% NPV for adverse cardiac events, which is the threshold most physicians consider acceptable.^19^, ^29^, ^33^, ^34^, ^35^ This includes the primary analysis of the US‐based STOP‐CP trial.^14^ Data from this subgroup analysis adds to that primary analysis by demonstrating that the algorithm failed to achieve an NPV ≥99% for 30‐day cardiac death or MI or 30‐day MACE among any sex or race subgroup. This suggests the hs‐cTnT ESC 0/1‐h algorithm alone may not be able to adequately identify US ED patients with chest pain who are safe for discharge. However, when combined with the HEAR score, the hs‐cTnT ESC 0/1‐h algorithm demonstrated safe NPV for 30‐day cardiac death or MI.

Our results demonstrated that the hs‐cTnT ESC 0/1‐h algorithm placed more women into the rule‐out zone compared to men. This is consistent with a prior subgroup analysis of the hs‐cTnT 0/1‐h algorithm in European and US cohorts.^6^, ^7^, ^12^, ^35^ Sex‐based differences in efficacy may be explained by sex‐specific differences in normal troponin ranges and thresholds for MI diagnosis. Previous evidence suggests that normal troponin concentrations are lower among women compared to men, possibly due to differences in left ventricular mass.^15^, ^36^, ^37^ The Fourth Universal Definition of Myocardial Infarction recommends using sex‐specific troponin cutoffs. When used, sex‐specific hs‐cTnT 99th percentile URL cutoffs increase the diagnosis of MI among women.^15^, ^26^ Kimenai et al. found that at given levels of elevated hs‐cTnT, women had a higher risk of cardiovascular events than men, which may explain why we found a higher point estimate odds of 30‐day cardiac death or MI for women in the rule‐in group compared to men.^38^ We did not observe increased rates of 30‐day cardiac death or MI or MACE for women relative to men in the rule‐out zone, but the study may have been underpowered to detect this difference. The higher proportion of 30‐day cardiac death or MI among women in the rule‐in zone compared to men suggests sex‐specific rule‐in hs‐cTnT thresholds may be needed for chest pain diagnostic pathways and should be a point of future study.

We found no significant between race and ESC 0/1‐h algorithm for 30‐day cardiac death or MI, with white patients in each ESC 0/1‐h algorithm zone having higher odds of 30‐day cardiac death or MI compared to nonwhite patients. However, despite a significantly higher proportion of nonwhite patients placed in the rule‐in zone compared to white patients, nonwhite patients had significantly lower odds of 30‐day cardiac death or MI compared to white patients in the rule‐in zone. This suggests that the use of hs‐cTnT ESC 0/1‐h algorithm may lead to disproportionate over‐testing among nonwhite patients. Of note, the finding of nonwhite patients having a lower rate of cardiovascular death is discordant to the rates typically found in the literature.^39^, ^40^ The underlying mechanisms for this are unclear and warrant further study but may be related to unmeasured social determinants of health.^39^ In addition, future studies are needed to determine if integrating individualized hs‐cTn cutoffs based on race, social determinants, or other variables into a 0/1‐h algorithm reduces over‐classification of patients into the rule‐in zone.

## 5 LIMITATIONS

Although this study was conducted at eight US EDs, these were mostly academic sites, which limits generalizability to other care settings. Informed consent was required to participate in STOP‐CP, resulting in possible selection bias. Lost to follow‐up rate was <4% (56 out of 1442); however, a sensitivity analysis imputing events for patients lost to follow‐up detailed in the primary manuscript of this study did not change the main results.^12^ Sex and race were determined by patient self‐reporting and medical record review which may have led to misclassification bias. Ethnicity was not included in this analysis. The 30‐day cardiac death or MI and MACE rates in STOP‐CP are higher than in previous US cohorts, and this increased prevalence may impact NPV and PPV.^12^, ^19^ As required by the Food and Drug Administration, the lowest reportable value for the hs‐cTnT assay was 6 ng/L, which is different than the ESC 0/1‐h algorithm rule‐out threshold hs‐cTnT value of 5 ng/L used outside of the United States. Our prior analyses suggest that this change has a minimal impact on the performance of the hs‐cTnT ESC 0/1‐h algorithm.^14^ However, it may affect the generalizability of these results outside of the United States. This study used only the Roche hs‐cTnT assay. Therefore, these conclusions cannot be applied to ESC 0/1‐h hs‐cTnI algorithm derivations. Adjudication was performed in the context of clinical use of contemporary troponin assays (Table S1). However, when adjudication was repeated using hs‐cTnT there was no substantive change.^14^ This pre‐planned subgroup analysis was designed to evaluate the performance of the existing ESC 0/1‐h hs‐cTnI algorithm among sex and race subgroups and not to seek optimal sex or race‐specific hs‐cTnI cutoffs or develop a new algorithm. Finally, this study was observational and as such, the ESC 0/1‐h hs‐cTnT algorithm was not used to guide patient care.

## 6 CONCLUSIONS

In this multisite, prospective US cohort study, the hs‐cTnT ESC 0/1‐h algorithm did not achieve an NPV ≥ 99% in any sex or race subgroup. Efficacy was significantly higher for women compared to men. More nonwhite patients were classified into the rule‐in zone, though they had significantly lower adjusted odds of 30‐day cardiac death or MI compared to white patients in the rule‐in zone. When the HEAR score and hs‐cTnT ESC 0/1‐h algorithm were combined, NPV ≥ 99% for 30‐day cardiac death or MI was achieved in all subgroups. Future studies should assess the integration of individualized hs‐cTnT thresholds to optimize risk stratification of ED patients with chest pain.

## CONFLICT OF INTEREST STATEMENT

Dr. Supples receives funding from the NIH (UL1TR001420) and the National Foundation of Emergency Medicine. Dr. O'Neill receives funding/support from Cytovale, Wake Forest CTSI, and the National Center for the Advancement of Translational Sciences. Dr. Ashburn receives funding from NHLBI (T32HL076132). Dr. Snavely receives funding from Abbott and HRSA (1H2ARH399760100). Dr. Stopyra receives research funding from NCATS/NIH (KL2TR001421), HRSA (H2ARH39976‐01‐00), Roche Diagnostics, Abbott Laboratories, Pathfast, Genetesis, Cytovale, Forest Devices, Vifor Pharma, and Chiesi Farmaceutici. Dr. Mahler receives funding/support from Roche Diagnostics, Abbott Laboratories, Ortho Clinical Diagnostics, Siemens, Grifols, Pathfast, Quidel, Genetesis, Cytovale, and HRSA (1H2ARH399760100). He is a consultant for Roche, Quidel, Abbott, Genetesis, Inflammatix, Radiometer, and Amgen and the Chief Medical Officer for Impathiq Inc. Dr. Allen receives research funding/support from Roche Diagnostics, Siemens Healthineers, and Beckman Coulter. He is a consultant for Roche Diagnostics. Dr. Nowak receives research funding/support from Roche Diagnostics, Beckmann Coulter, Siemens, Abbott Diagnostics, and Ortho Clinical Diagnostics. He is a consultant for Roche Diagnostics, Beckman Coulter, Siemens, Abbott Diagnostics, and Ortho Clinical Diagnostics. Dr Mumma has research support from the NIH (5K08HL130546) and Roche Diagnostics. Dr Christenson is a consultant for and receives funding/support from Roche Diagnostics, Siemens Healthineers, Beckman Coulter Diagnostics, Becton Dickinson and Co., Quidel Corp, and Sphingotec GMBH. Dr. Wilkerson receives research support from Regeneron, Eli Lilly, Cepheid, CoapTech, Global Blood Therapeutics, Novartis, EndPoint Health, Roche, Vapotherm, and Eldon. He has received research support from the National Heart, Lung, and Blood Institute (No. U24HL137907) and the National Institute for Diabetes and Digestive and Kidney Diseases (No. R44DK115325). He has been a contracted author for Elsevier Publishing and Relias Learning LLC. The other authors report no conflicts.

## Supporting information

Supporting information.

## Data Availability

Research data are not shared. Due to the nature of the research, supporting data are not available.
