# Supplementary material for: Sex and race differences in the performance of the European Society of Cardiology 0/1‐h algorithm with high‐sensitivity troponin T
Source: Clin Cardiol. 2023 Dec 13;47(2):e24199. doi: 10.1002/clc.24199 (PMC10823440; doi:10.1002/clc.24199)
Supplement: Supplementary file 1 — Supporting information. [file CLC-47-e24199-s001.docx]

**Supplemental Table 1.** Contemporary troponin analytical data by clinical site. Adapted from the IFCC table (http://www.ifcc.org/media/276664/IFCC%20Troponin%20Tables%20ug_L_DRAFT%20Update%20NOVEMBER%202014.pdf).

| **Clinical Site** | **Contemporary Troponin Assay** | **99^th^ Percentile Upper Reference Limit** | **10% Coefficient of Variation** |
| --- | --- | --- | --- |
| **University of Florida** | Roche Trop T | 0.01 ng/mL | 0.03 ng/mL |
|  | Beckman Coulter Trop I | 0.04 ng/mL | 0.06 ng/mL |
| **Wake Forest University** | Siemens TnI Ultra | 0.04 ng/mL | 0.03 ng/mL |
| **Henry Ford Health System** | Siemens TnI Ultra | 0.04 ng/mL | 0.03 ng/mL |
| **University of Maryland** |  | | |
| St. Joseph Medical Center | Ortho Clinical Diagnostics cTnI | 0.034 ng/mL | 0.034 ng/mL |
| University Medical Center | Ortho Clinical Diagnostics cTnI  Pathfast cTnI | 0.034 ng/mL  0.029 ng/mL | 0.034 ng/mL  0.036 ng/mL |
| Baltimore Washington  Medical Center | Beckman Coulter Trop I | 0.04 ng/mL | 0.06 ng/mL |
| **University of California-Davis** | Siemens TnI Ultra | 0.04 ng/mL | 0.03 ng/mL |
| **University of Utah** | Abbott Architect | 0.028 ng/mL | 0.032 ng/mL |

**Supplemental Table 2.** Demographic, hs-cTnI, HEAR score and known CAD description of each patient with cardiac death or MI within 30 days who was placed into the rule-out zone by the ESC 0/1 hs-cTnT algorithm.

| **Age** | **Sex** | **Race** | **0-hour hs-cTnI (ng/dL)** | **1-hour hs-cTnI (ng/dL)** | **HEAR Score** | **Known CAD** |
| --- | --- | --- | --- | --- | --- | --- |
| 65 | Male | White | 11 | 10 | 4 | Yes |
| 55 | Male | Black | 7 | 7 | 5 | No |
| 64 | Male | White | 10 | 11 | 2 | No |
| 56 | Male | White | 11 | 11 | 5 | Yes |
| 76 | Female | White | 9 | 7 | 4 | Yes |
| 54 | Female | Black | 10 | 12 | 3 | No |
| 69 | Female | White | 4 | 3 | 5 | No |
| 47 | Female | Black | 3 | 2 | 6 | No |
| 54 | Male | White | 10 | 10 | 4 | No |
| 62 | Male | Black | 11 | 11 | 4 | No |
| 70 | Male | White | 6 | 6 | 6 | Yes |
| 62 | Female | White | 5 | 4 | 4 | No |
| 45 | Male | White | 5 | 5 | 3 | Yes |

**Supplemental Table 3**: Index and 30-day events including all-cause mortality.

| **RULE-OUT** | Female n = 436 ,n (%) | Male n = 385, n (%) | Non-white n = 331, n (%) | White n = 490, n (%) | Total n = 821, n (%) |
| --- | --- | --- | --- | --- | --- |
| **Index** | 1 (0.2) | 0 (0) | 0 (0) | 1 (0.2) | 1 (0.1) |
| All-Cause Death | 1 (0.2) | 0 (0) | 0 (0) | 1 (0.2) | 1 (0.1) |
| MI | 4 (0.9) | 4 (1) | 2 (0.6) | 6 (1.2) | 8 (1) |
| All-Cause Death or MI | 5 (1.1) | 4 (1) | 2 (0.6) | 7 (1.4) | 9 (1.1) |
| **30-day (excludes index)** |  |  |  |  |  |
| All-Cause Death | 0 (0) | 0 (0) | 0 (0) | 0 (0) | 0 (0) |
| MI | 0 (0) | 4 (1) | 2 (0.6) | 2 (0.4) | 4 (0.5) |
| All-Cause Death or MI | 0 (0) | 6 (1.6) | 3 (0.9) | 3 (0.6) | 6 (0.7) |
|  |  |  |  |  |  |
|  |  |  |  |  |  |
| **OBSERVATION** | Female n = 159, n (%) | Male n = 252, n (%) | Non-white n = 172, n (%) | White n = 239, n (%) | Total N = 411, n (%) |
| **Index** |  |  |  |  |  |
| All-Cause Death | 0 (0) | 0 (0) | 0 (0) | 0 (0) | 0 (0) |
| MI | (0) | (0) | (0) | (0) | 0 (0) |
| All-Cause Death or MI | 13 (8.2) | 38 (15.1) | 14 (8.1) | 37 (15.5) | 51 (12.4) |
| **30-day (excludes index)** |  |  |  |  |  |
| All-Cause Death | 2 (1.3) | 5 (2) | 1 (0.6) | 6 (2.5) | 7 (1.7) |
| MI | 2 (1.3) | 6 (2.4) | 3 (1.7) | 5 (2.1) | 8 (1.9) |
| All-Cause Death or MI | 4 (2.5) | 10 (4) | 4 (2.3) | 10 (4.2) | 14 (3.4) |
|  |  |  |  |  |  |
|  |  |  |  |  |  |
| **RULE-IN** | Female n = 57, n (%) | Male n = 133, n (%) | Non-white n = 93, n (%) | White n = 97, n (%) | Total N = 190, n (%) |
| **Index** |  |  |  |  |  |
| All-Cause Death | 0 (0) | 1 (0.8) | 0 (0) | 1 (1) | 1 (0.5) |
| MI | 35 (61.4) | 70 (52.6) | 44 (47.3) | 61 (62.9) | 105 (55.3) |
| All-Cause Death or MI | 35 (61.4) | 70 (52.6) | 44 (47.3) | 61 (62.9) | 105 (55.3) |
| **30-day (excludes index)** |  |  |  |  |  |
| All-Cause Death | 0 (0) | 6 (4.5) | 3 (3.2) | 3 (3.1) | 6 (3.2) |
| MI | 5 (8.8) | 7 (5.3) | 6 (6.5) | 6 (6.2) | 12 (6.3) |
| All-Cause Death or MI | 5 (8.8) | 12 (9) | 8 (8.6) | 9 (9.3) | 17 (8.9) |

Supplemental Table 4. Test characteristics of ESC 0/1

|  | | **30-day Cardiac Death or MI** | | | |
| --- | --- | --- | --- | --- | --- |
|  |  | **Females** | **Males** | **Non-whites** | **Whites** |
| **Rule-Out** | Sensitivity (95%CI) | 91.2 (80.7-97.1) | 93.7 (87.9-97.2) | 94.0 (85.4-98.3) | 92.2 (85.8-96.4) |
|  | NPV (95%CI) | 98.9 (97.3-99.6) | 97.9 (95.9-99.1) | 98.8 (96.9-99.7) | 98.2 (96.5-99.2) |
|  | -LR (95%CI) | 0.12 (0.05-0.28) | 0.11 (0.06-0.21) | 0.10 (0.04-0.25) | 0.12 (0.06-0.22) |
| **Rule-In** | Specificity (95%CI) | 96.5 (94.7-97.8) | 91.0 (88.5-93.1) | 91.1 (88.4-93.4) | 95.5 (93.7-96.9) |
|  | PPV (95%CI) | 63.2 (49.3-75.6) | 56.4 (47.5-65.0) | 49.5 (38.9-60.0) | 67.0 (56.7-76.2) |
|  | +LR (95%CI) | 17.9 (11.3-28.5) | 6.6 (5.0-8.8) | 7.7 (5.6-10.6) | 12.4 (8.5-18.1) |
|  | | **30-day MACE** | | | |
|  |  | **Females** | **Males** | **Non-whites** | **Whites** |
| **Rule-Out** | Sensitivity (95%CI) | 87.3 (76.5-94.4) | 89.3 (82.9-93.9) | 90.1 (80.7-95.9) | 87.9 (81.1-92.9) |
|  | NPV (95%CI) | 98.2 (96.4-99.2) | 96.1 (93.7-97.8) | 97.9 (95.7-99.1) | 96.7 (94.8-98.1) |
|  | -LR (95%CI) | 0.18 (0.09-0.34) | 0.18 (0.11-0.30) | 0.16 (0.08-0.32) | 0.18 (0.11-0.28) |
| **Rule-In** | Specificity (95%CI) | 96.4 (94.6-97.8) | 90.8 (88.3-92.9) | 91.0 (88.3-93.3) | 95.4 (93.6-96.8) |
|  | PPV (95%CI) | 63.2 (49.3-75.6) | 56.4 (47.5-65.0) | 49.5 (38.9-60.0) | 67.0 (56.7-76.2) |
|  | +LR (95%CI) | 16.0 (10.0-25.7) | 5.8 (4.4-7.8) | 7.2 (5.2-10.0) | 10.7 (7.3-15.6) |

**Supplemental Table 5:** Test characteristics for 30-day all-cause mortality or myocardial infarction (inclusive of index events) for ESC 0/1 and ECC 0/1 combined with HEAR score.

|  | | **ESC 0/1** | | | |
| --- | --- | --- | --- | --- | --- |
|  |  | **Females** | **Males** | **Non-whites** | **Whites** |
| **Rule-Out** | Sensitivity (95%CI) | 91.4 (81.0-97.1) | 92.4 (86.4-96.3) | 92.8 (83.9-97.6) | 91.7 (85.2-95.9) |
|  | NPV (95%CI) | 98.9 (97.3-99.6) | 97.4 (95.3-98.7) | 98.5 (96.5-99.5) | 98.0 (96.3-99.0) |
|  | -LR (95%CI) | 0.12 (0.05-0.28) | 0.13 (0.07-0.24) | 0.12 (0.05-0.27) | 0.12 (0.07-0.22) |
| **Rule-In** | Specificity (95%CI) | 96.5 (94.6-97.8) | 90.9 (88.4-93.0) | 91.1 (88.3-93.4) | 95.5 (93.7-96.9) |
|  | PPV (95%CI) | 63.2 (49.3-75.6) | 56.4 (47.5-65.0) | 49.5 (38.9-60.0) | 67.0 (56.7-76.2) |
|  | +LR (95%CI) | 17.6 (11.0-28.0) | 6.3 (4.7-8.4) | 7.5 (5.4-10.3) | 12.0 (8.2-17.4) |
|  | | **ESC 0/1 + HEAR Score** | | | |
|  |  | **Females (n=652)** | **Males (n=770)** | **Non-whites (n=596)** | **Whites (n=826)** |
| **Rule-Out** | Sensitivity (95%CI) | 98.3 (90.8-100.0) | 96.9 (92.4-99.2) | 97.1 (89.9-99.6) | 97.5 (92.9-99.5) |
|  | NPV (95%CI) | 99.6 (97.6-100.0) | 98.1 (95.1-99.5) | 98.9 (96.2-99.9) | 98.8 (96.5-99.9) |
|  | -LR (95%CI) | 0.04 (0.01-0.31) | 0.10 (0.04-0.26) | 0.08 (0.02-0.32) | 0.07 (0.02-0.22) |
| **Rule-In** | Specificity (95%CI) | 93.6 (91.3-95.4) | 88.6 (85.9-90.9) | 88.8 (85.8-91.4) | 92.6 (90.5-94.5) |
|  | PPV (95%CI) | 48.6 (36.9-60.6) | 51.6 (43.4-59.9) | 43.8 (34.1-53.8) | 56.7 (48.3-65.7) |
|  | +LR (95%CI) | 9.7 (6.7-14.0) | 5.2 (4.0-6.7) | 6.0 (4.4-8.0) | 7.7 (5.7-10.4) |

**Supplemental Table 6.** Adjusted odds ratios (aOR) for safety events among ESC 0/1-hour hs-cTnT algorithm patients who were male vs. female and white vs. non-white.

| **Rule-Out** | **aOR (95%CI)^a^ Sex** | **aOR (95%CI)^a^ Race** |
| --- | --- | --- |
| Index Cardiac Death or MI | 0.82 (0.22-3.10) | 2.17 (0.45-10.5) |
| Index MACE | 1.29 (0.47-3.52) | 2.71 (0.76-9.67) |
| 30-day Cardiac Death or MI | 1.67 (0.54-5.18) | 1.41 (0.43-4.66) |
| 30-day MACE | 1.85 (0.77-4.47) | 1.47 (0.59-3.64) |
| **Observation** |  |  |
| Index Cardiac Death or MI | 1.86 (0.94-3.68) | 1.81 (0.92-3.57) |
| Index MACE | 2.08 (1.06-4.09) | 2.05 (1.05-4.00) |
| 30-day Cardiac Death or MI | 1.79 (0.95-3.37) | 1.75 (0.93-3.27) |
| 30-day MACE | 1.72 (0.95-3.12) | 2.15 (1.18-3.94) |
| **Rule-In** |  |  |
| Index Cardiac Death or MI | 0.51 (0.25-1.05) | 1.62 (0.85-3.10) |
| Index MACE | 0.49 (0.24-1.01) | 1.54 (0.81-2.95) |
| 30-day Cardiac Death or MI | 0.55 (0.27-1.12) | 1.73 (0.90-3.33) |
| 30-day MACE | 0.52 (0.26-1.08) | 1.73 (0.90-3.33) |

MI – myocardial infarction, MACE – major adverse cardiovascular event, CAD – coronary artery disease

^a^ Adjustment was made for age, sex, race, hypertension, diabetes, hyperlipidemia, obesity, current smoking, prior stroke, peripheral vascular disease, and end stage renal disease as well as the interaction between ESC 0/1-hour classification and known CAD.

**Supplemental Table 7:** Test characteristics of ESC 0/1 combined with HEAR score.

|  | | **30-day Cardiac Death or MI** | | | |
| --- | --- | --- | --- | --- | --- |
|  |  | **Females (n=652)** | **Males (n=770)** | **Non-whites (n=596)** | **Whites (n=826)** |
| **Rule-Out** | Frequency (%) | 233 (35.7%) | 206 (26.8%) | 188 (31.5%) | 251 (30.4%) |
|  | Sensitivity (95%CI) | 98.2 (90.6-100) | 98.4 (94.4-99.8) | 98.5 (92.0-100) | 98.3 (93.9-99.8) |
|  | NPV (95%CI) | 99.6 (97.6-100) | 99.0 (96.5-99.9) | 99.5 (97.1-100) | 99.2 (97.2-99.9) |
|  | -LR (95%CI) | 0.05 (0.01-0.3) | 0.05 (0.01-0.2) | 0.04 (0.01-0.3) | 0.05 (0.01-0.2) |
| **Rule-In** | Frequency (%) | 74 (11.3%) | 151 (19.6%) | 105 (17.6%) | 120 (14.5%) |
|  | Specificity (95%CI) | 93.6 (91.3-95.4) | 88.7 (86.0-91.0) | 88.8 (85.8-91.4) | 92.7 (90.5-94.5) |
|  | PPV (95%CI) | 48.6 (36.9-60.6) | 51.7 (43.4-59.9) | 43.8 (34.1-53.8) | 56.7 (47.3-65.7) |
|  | +LR (95%CI) | 9.9 (6.6-14.3) | 5.5 (4.2-7.1) | 6.2 (4.6-8.2) | 8.0 (5.9-10.8) |
|  | | **30-day MACE** | | | |
|  |  | **Females (n=652)** | **Males (n=770)** | **Non-whites (n=596)** | **Whites (n=826)** |
| **Rule-Out** | Frequency (%) | 233 (35.7%) | 206 (26.8%) | 188 (31.5%) | 251 (30.4%) |
|  | Sensitivity (95%CI) | 95.2 (86.7-99.0) | 97.1 (92.8-99.2) | 97.2 (90.2-99.7) | 96.2 (91.4-98.8) |
|  | NPV (95%CI) | 98.7 (96.3-99.7) | 98.1 (95.1-99.5) | 98.9 (96.2-99.9) | 98.0 (95.4-99.4) |
|  | -LR (95%CI) | 0.12 (0.04-0.4) | 0.09 (0.03-0.24) | 0.08 (0.02-0.3) | 0.11(0.05-0.3) |
| **Rule-In** | Frequency (%) | 74 (11.3%) | 151 (19.6%) | 188 (31.5%) | 251 (30.4%) |
|  | Specificity (95%CI) | 93.5 (91.3-95.4) | 88.7 (86.0-91.1) | 88.8 (85.7-91.3) | 92.8 (90.6-94.6) |
|  | PPV (95%CI) | 48.6 (36.9-60.6) | 53.0 (44.7-61.1) | 43.8 (34.1-53.8) | 58.3 (49.0-67.3) |
|  | +LR (95%CI) | 8.9 (6.1-12.9) | 5.1 (3.9-6.6) | 5.8 (4.3-7.7) | 7.4 (5.4-10.1) |

**Supplemental Figures**


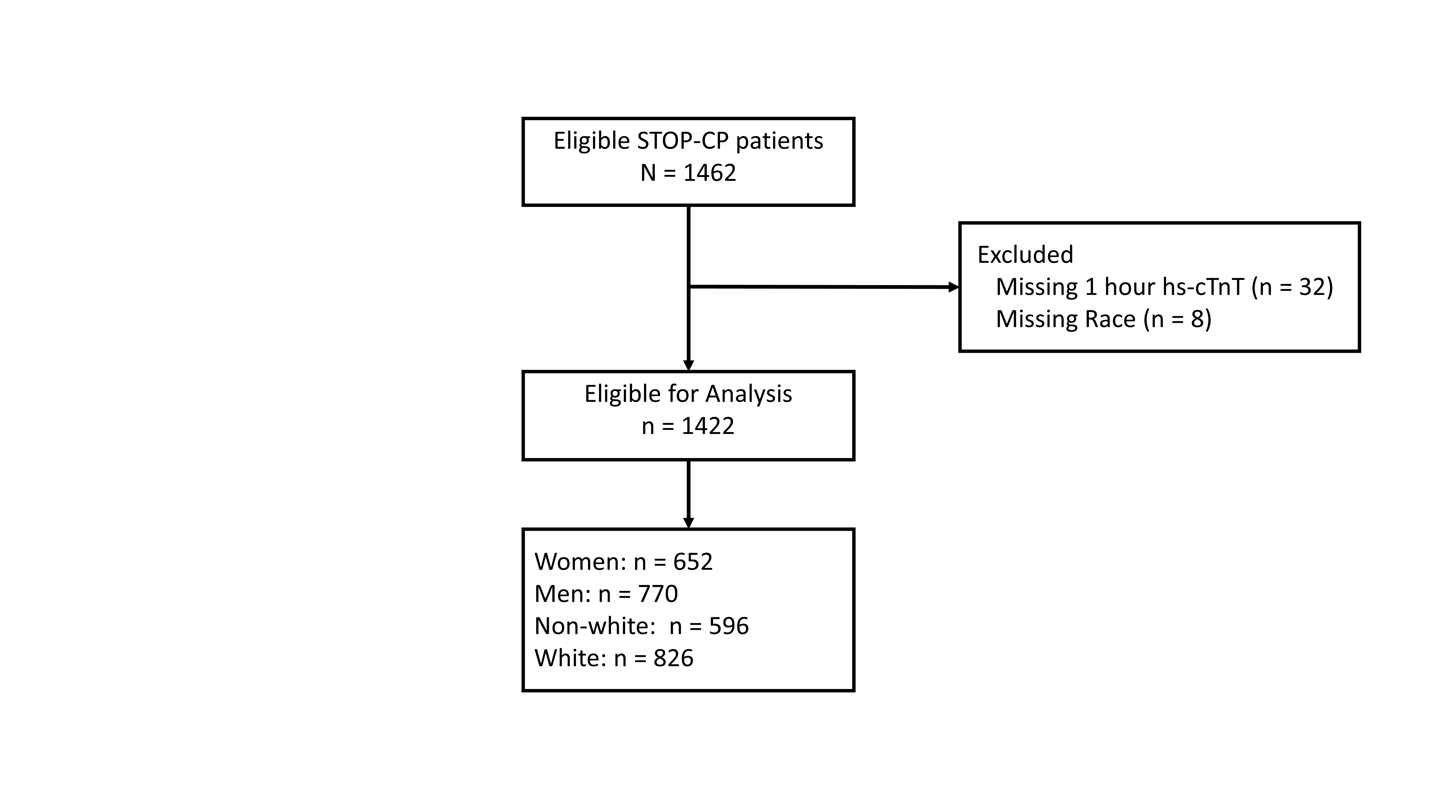


Supplemental Figure 1. Patient flow diagram.


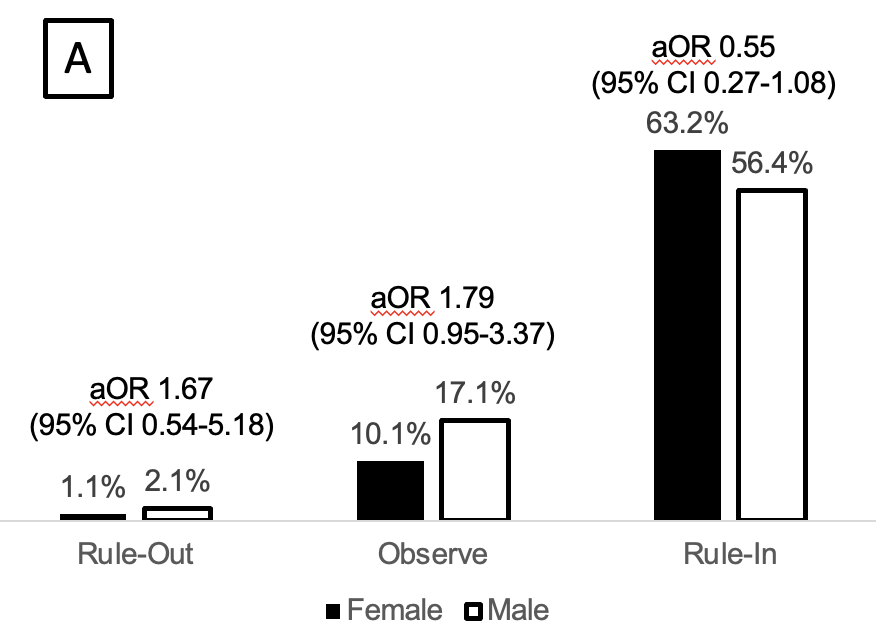


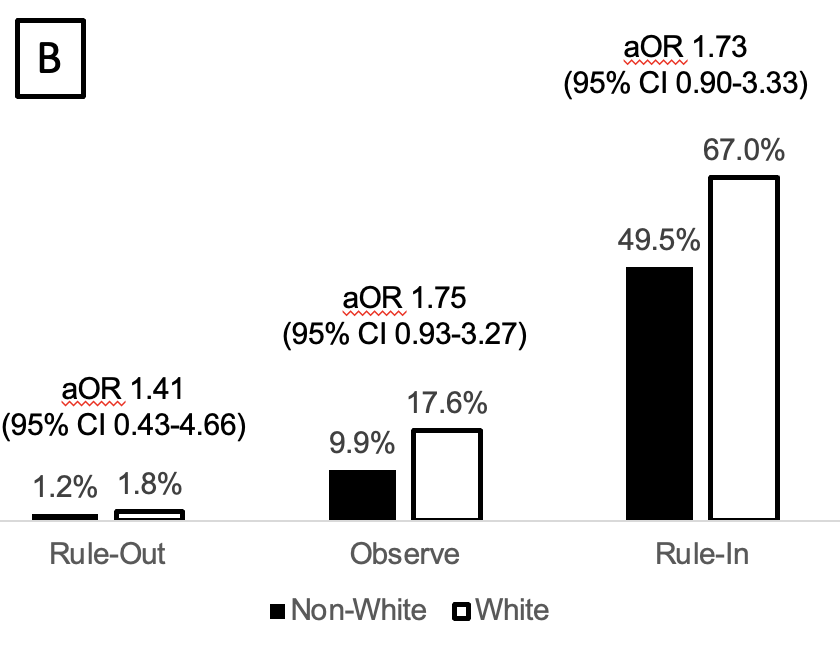


**Supplemental Figure 2A-B.** Proportion of patients with 30-day cardiac death or MI by ESC0/1 category. A – Sex, B – Race

ACS – acute coronary syndrome, CAD – coronary artery disease, ED – emergency department; MI – myocardial infarction; NPV – negative predictive value, PPV – positive predictive value, LR – likelihood ratio, aOR – adjusted odds ratio.
